# BMI and dissatisfaction with life: contextual factors and socioemotional costs of obesity

**DOI:** 10.1007/s11136-021-02912-3

**Published:** 2021-06-21

**Authors:** Ewa Jarosz, Alexi Gugushvili

**Affiliations:** 1 Institute of Philosophy and Sociology, Polish Academy of Sciences, Nowy Świat 72, 00-330 Warsaw, Poland; 2 Department of Sociology and Human Geography, University of Oslo, Moltke Moes vei 31, Harriet Holters hus, 0851 Oslo, Norway

**Keywords:** BMI, Life satisfaction, Social comparison, Life in Transition Survey, Body weight

## Abstract

**Purpose:**

In this study, we investigate whether individuals’ BMI categories are associated with being dissatisfied with one’s life, how this association is affected by the social comparison that individuals make, and what the role of the overall BMI levels in this process is.

**Methods:**

We use data for 21,577 men and 27,415 women, collected in 2016 by the European Bank for Reconstruction and Development, from 34 countries in Europe, the Middle East, and Central Asia. To understand the moderating effect of contextual environment, we use multilevel mixed effect logistic regression models and data for national, regional, and cohort-specific BMI levels.

**Result:**

We find that the association of BMI and dissatisfaction with life differs by gender, with overweight men being less likely to be dissatisfied with life than men with normal weight and obese women being more likely to be dissatisfied with life compared to women with normal weight. For contextual effects, we find that obese women in regions with low BMI levels are more likely to be dissatisfied with life. The effect of obesity on female life dissatisfaction is not observed in regions with high BMI levels. As for men, regional BMI levels affect the levels of life dissatisfaction but only for underweight men.

**Conclusions:**

Our study adds additional nuance to the quality-of-life research by showing that the association between BMI and decreased life satisfaction is, at least partially, moderated by the contextual environment, and that the character of these effects differs by gender.

**Supplementary Information:**

The online version contains supplementary material available at 10.1007/s11136-021-02912-3.

## Introduction

Body mass index (BMI) is an anthropometric measure that has associations with nearly all areas of an individual’s physical functioning. Most commonly, BMI has been investigated in relation to various health outcomes such as self-rated health [1], morbidity [2], and mortality [3]. BMI is also associated with a broad set of behaviours and practices including daily activities [4], social life [5], mating [6], and labour market performance [7]. As BMI is linked to many aspects of an individual’s life, it is likely that it has direct or indirect effects on an individual’s overall life satisfaction (LS). Indeed, studies have found that those who are overweight or obese report decreased life satisfaction compared to people with BMI scores in the range which is considered as normal [8–10]. Obese individuals are also at a higher risk of depression [11], having low self-esteem [12], and are more likely to experience discrimination due to their weight [13].

Although specific causal channels linking BMI and LS are still being debated, it is likely that both individual and social characteristics affect this association. A number of studies have reported that LS is more likely to be influenced by the perceived weight status than with the actual BMI [14, 15]. How the actual or perceived weight affects LS also depends on the individuals’ gender [16, 17], which in turn is related to different levels of obesity stigmatisation among men and women [18]. Yet, for certain sociodemographic groups, such as older adults, no systematic and consistent associations were found between BMI and LS [19, 20]. That suggests that certain individual or contextual characteristics moderate the effect of BMI on LS. There is also evidence that the individuals’ own weight estimates are affected by the average weight of their age- and gender-specific reference groups [21]. The role of social comparison is highlighted in studies which show that an individuals’ BMI is associated with the BMI of other adults within their close social network [22]. Therefore, an individuals’ group of reference in social comparison might be a factor moderating the association between BMI and LS. To our knowledge, there are virtually no studies analysing this specific research question.

We can also assume that contextual environment shapes individuals’ attitudes towards their own and others’ physical traits, yet the environment in which individuals live varies across countries and over time. The prevalence of obesity, both in Europe and globally, has increased substantially over the past decades [23, 24]. As adiposity becomes more prevalent in a society, being overweight or obese is more likely to be perceived as the new “normal weight” [15]. According to the social comparison theory, people who are overweight and obese are less likely to stand out when the overall BMI levels are high. However, research on how the effect of BMI on LS is shaped by the social context is limited and the evidence is mixed. Small sample studies have reported no association between one’s own weight and obesity rates among the social comparison reference group members [25]. Conversely, some large-scale studies suggest that in areas with high levels of BMI, the negative effect of obesity on LS is weaker, and that this association differs by gender [17].

In this study, we focus on the socioemotional costs of having other than normal BMI. We explore the effect of different BMI categories on being dissatisfied with life, taking into account the individual and contextual characteristics that might affect this relationship. In particular, we aim to investigate whether a person’s reference group for social comparison, and obesity rates in a given population affect the relationship between BMI and dissatisfaction with life. One of the methodological challenges in identifying statistically significant associations and pinpointing a direction of causation between outcome and explanatory variables is that being dissatisfied with life might itself, either directly or indirectly, affect the individuals’ belonging to certain BMI categories. We mitigate this concern in our empirical analyses by employing the treatment estimators’ approach for observational data in which the BMI category of individuals is predicted, among other factors, by the number of hours that individuals spend watching television.

## Methods

### Dataset

We analyse data from the Life in Transition Survey (LITS) collected in 2016 by the European Bank for Reconstruction and Development (EBRD) [26], from 34 countries in Europe, the Middle East, and Central Asia (list of countries and sociodemographic composition of samples is given in Table S1 in the supplementary materials). LITS has been recently used in comparative health and wellbeing research [27–29], including in studies on individuals’ anthropometric measures and their effects on wellbeing [30, 31]. LITS ensures the national representativeness of the collected data by using a multi-stage random probability stratified clustered sampling. After list-wise deletion of observations with missing information (4.3% of the total sample), 21,577 males and 27,415 females were available for our analysis. The main results of the study, however, are essentially unaffected when missing data for 2,214 individuals are also generated via Multiple Imputation using Chained Equations (MICE) approach (see Table S2 in the supplementary materials).

### Dissatisfaction with life

Individuals’ LS in LITS is measured using the Likert scale answer options from “strongly disagree” = 1 to “strongly agree” = 5 for the following survey statement: “all things considered, I am satisfied with my life now” (mean 3.2, SD 1.1). This survey item captures less variation of individuals’ LS than the more conventionally used larger response scale range with integer values from “dissatisfied”  = 1 to “satisfied” = 10. Nevertheless, the measure we use has been successfully employed in comparative wellbeing research in the countries included in our study [32–34]. As we are primarily interested in whether individuals’ BMI predicts dissatisfaction with life and under what circumstances, we dichotomize the dependant variable by assigning to it a value of 1 if respondents strongly disagree or disagree that they are satisfied with their lives.

### BMI categories

We created the BMI categories using individuals’ self-reported weight and height information. By comparing actual and self-reported anthropometric measures, the previous research demonstrates that self-reported estimates are good proxies for actual height and weight [35, 36]. After dividing individuals’ weight in kilogrammes by their height in metres squared, we classified the derived BMI scores as underweight (below 18.5, men 0.9%, women 2.7%), normal weight (18.5–24.9, men 33.6%, women 39.2%), overweight (25.0–29.9, men 39.7%, women 28.8%), and obese (30.0 and above, men 25.7%, women 29.3%).

### Predictors of dissatisfaction with life

In our multivariable models, we include a number of predictors in order to account for possible confounders of the association between BMI and dissatisfaction with life. In addition to fitting separate models by gender, we considered sociodemographic measures of age, whether individuals lived in an urban or rural part of the country, and their marital status (never married, married, widowed, and separated/divorced). We differentiated individuals’ educational attainment by primary, secondary, and tertiary levels, and their labour market status into those who had never worked, who were unemployed, and who were employed at the time of the interview.

Individuals’ material deprivation was operationalised by the number of following items which their households could not afford to have (1) telephone, including mobile phone; (2) colour TV set; (3) computer, laptop, or tablet; (4) washing machine; (5) car, including company car used for private purposes; (6) bicycle; and (7) motorcycle. The created variable takes a value of 0 if respondents’ households are not deprived at all and a value of 7 if they are deprived of all the listed items [37]. We operationalised subjective socioeconomic status by respondents' self-placement on an abstract hierarchical ladder in which the first step included the poorest 10% of households and the tenth step represented the richest 10% of households in countries where the individuals lived [38]. To account for material conditions directly affecting individuals’ wellbeing, we used the survey question asking if respondents’ households could afford the consumption of meat, chicken, fish, or a vegetarian equivalent each second day.

The potential effect of trust on life dissatisfaction was examined by asking about the extent to which respondents had trust in other people [39]. We coded this binary variable as 1 if individuals had a complete distrust in others. Dissatisfaction with life is known to be affected by the patterns of socialising which we operationalised by how often individuals met with their friends and family from outside the household. This variable varies from “never” = 1 to “on most days” = 5.

To account for the possible effect of social comparison and its moderating effect, we used the following LITS question: “When thinking of your current economic situation, which of these is most likely to be your benchmark?” All respondents had four answer categories to choose from “how your parents lived at your age”; “how your friends and neighbours live”; “how the domestic elite lives”; and “how people live in Western Europe”. Respondents could also select “no comparison group” [27, 40]. We separated the comparison group with friends and neighbours from other comparison groups in our variable specification. To account for the effect of BMI on dissatisfaction with life through health, we controlled for individuals’ self-rated health. We created a dummy variable with the value of 1 if individuals rated their health as “bad” or “very bad”. Table S3 in the supplementary materials shows the descriptive statistics of all individual-level predictors of dissatisfaction with life.

### Contextual factors

To account for the overall levels of BMI in countries where individuals reside and to test if contextual BMI characteristics moderate the effect of individual-level BMI categories, we used the mean levels of BMI for 2016 reported by the World Health Organisation [41]. Another variable that we used is the standard deviation on BMI scores within countries, which reflects how BMI scores vary within the considered nations. Furthermore, the social comparison of BMI levels might take place not across all individuals but particularly among those who belong to the same age group. This is why we generated cohort-specific BMI scores that were calculated specifically for those who had been born in the same decade in a particular country. At last, the national level of BMI scores might be too distant to adequately reflect individuals’ own surroundings. Therefore, we also calculated regional-level BMI scores for individuals who reside in the considered subnational territorial units given in LITS.

### Statistical analysis

After presenting age- and country-adjusted levels of dissatisfaction with life by BMI categories separately by men and women, we fit logistic regression models with the outcome measure on dissatisfaction with life. However, the conventional regression approach might produce biased estimates as individuals’ selection into different BMI categories is not adequately accounted for. Existing research suggests that the level of satisfaction with life can affect an individual’s diet, exercise patterns, and other characteristics which are known to cause changes in the BMI score, or, both BMI and being satisfied or dissatisfied with one’s life can be determined by other unobserved circumstances [42]. To mitigate this problem, we derive treatment effects from observational data by using inverse-probability-weighted regression adjustment (IPWRA) estimators [43]. IPWRA estimators use weighted regression coefficients to compute averages of treatment-level-predicted outcomes. The weights used to adjust regression coefficients are derived from inverse probabilities of treatment. In turn, inverse-probability weights are estimated separately from the parameters of the treatment model. After adjusting for likelihood of treatment, in our case being in different BMI categories, the contrasts of the averages in different treatment groups provide the estimated treatment effects.

For the treatment model, we use the subset of variables described above which consists of individuals’ age, settlement type, marital status, education, labour market status, material deprivation, the affordability of consumption of meat, chicken, fish, or vegetarian equivalent each second day, and subjective socioeconomic status. In addition, we add a variable to this list on the number of hours that individuals watched television each day prior to the interview. This measure is an important predictor of the BMI scores as it is an indicator of a sedentary lifestyle. Watching television for long hours has been shown to be associated with a higher BMI score [4], and this variable has also been used as an instrument to predict individuals BMI in recent studies [31, 44].

Treatment estimators allow us to test if the patterns observed in conventional regression settings also hold a more robust analytical design, but they are limited in terms of understanding how contextual environment related to national, regional, and cohort-specific BMI scores moderates the effect of BMI on dissatisfaction with life. For this purpose, we also employ multilevel mixed effects logistic regression models with random intercept and cross-level interaction terms. In this analytical framework, level 1 is composed of individuals, while level 2 is composed of countries where individuals reside.

## Results

### Bivariate associations between BMI and dissatisfaction with life

**Fig. 1:**
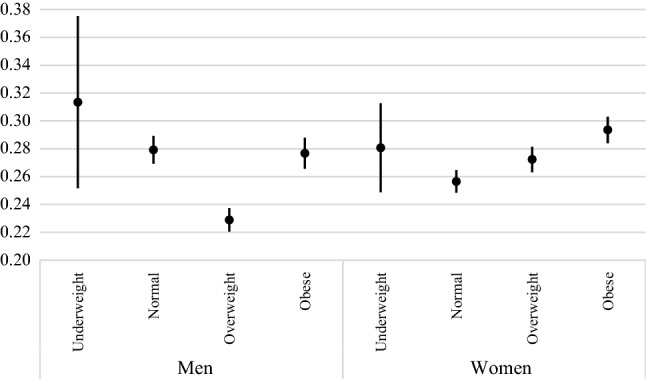
Prevalence of dissatisfaction with life by BMI in the pooled sample of 34 societies, predictive margins from age- and country-adjusted logistic regressions. *Source* Authors’ calculations based on data from LITS

Figure 1 shows men and women’s levels of dissatisfaction with life according to their BMI categories. The presented predictive margins account for the individuals’ age and country fixed effects and suggest that in the pooled sample of 34 countries, dissatisfaction with life is the lowest among overweight men. When compared with overweight men, obese men have higher levels of dissatisfaction with life. There is no significant difference between men with normal BMI scores and those who are obese. A different picture emerges for the sample of women where the lowest prevalence of dissatisfaction with life is observed for those who have normal BMI scores. The gradient in life dissatisfaction between all BMI categories is clearly visible among women with the prevalence of dissatisfaction with life being the highest for obese women.

### Is BMI linked with dissatisfaction with life in multivariable settings?

In Table 1, we see that the associations observed in Fig. 1 hold also when an extensive set of predictors of dissatisfaction with life is accounted for. Overweight men have odds ratios of 0.83 and 0.85 for life dissatisfaction, respectively, in Models 1 and 2 when compared with men with normal BMI scores. In Model 1, overweight and obese women have, respectively, odds ratios of 1.11 and 1.18 for dissatisfaction with life compared to women who are in the normal BMI category. The validity of fitted models can be observed by the results for other predictors of dissatisfaction with life. Age, settlement type, marital status, educational attainment, employment status, material deprivation, subjective and objective socioeconomic position, the patterns of socialisation, social trust, and self-rated health all significantly explain the variation in dissatisfaction with life in the expected direction. Comparing own socioeconomic status with those other than friends and neighbours is also linked with a higher chance of being dissatisfied with life.

**Table 1:** BMI and dissatisfaction with life in the pooled sample of 34 societies and odds ratios from logistic regressions

	Men				Women			
	Model 1		Model 2		Model 1		Model 2	
	β	(SE)	β	(SE)	β	(SE)	β	(SE)
Intercept	1.16	(0.25)	1.06	(0.25)	0.34***	(0.06)	0.38***	(0.08)
BMI								
Underweight	0.97	(0.17)	0.87	(0.16)	1.12	(0.11)	1.08	(0.10)
Normal	1.00	–	1.00	–	1.00	–	1.00	–
Overweight	0.83***	(0.03)	0.85***	(0.04)	1.11**	(0.04)	1.11**	(0.04)
Obese	1.06	(0.05)	1.06	(0.05)	1.18***	(0.05)	1.14***	(0.05)
Socio-demographics								
Age	1.04***	(0.01)	1.04***	(0.01)	1.05***	(0.01)	1.05***	(0.01)
Age^2^	1.00***	(0.00)	1.00***	(0.00)	1.00***	(0.00)	1.00***	(0.00)
Settlement								
Rural	1.00	–	1.00	–	1.00	–	1.00	–
Urban	0.91**	(0.03)	0.91*	(0.03)	0.91**	(0.03)	0.92*	(0.03)
Marital status								
Single	0.71***	(0.06)	0.73***	(0.06)	0.88*	(0.06)	0.86*	(0.06)
Married	0.64***	(0.04)	0.67***	(0.05)	0.72***	(0.04)	0.72***	(0.04)
Widowed	0.77**	(0.07)	0.79*	(0.08)	0.88*	(0.05)	0.89	(0.06)
Divorced	1.00	–	1.00	–	1.00	–	1.00	–
Education								
Primary	1.00	–	1.00	–	1.00	–	1.00	–
Secondary	0.87**	(0.04)	0.88**	(0.04)	0.93	(0.04)	0.96	(0.04)
Tertiary	0.74***	(0.04)	0.76***	(0.04)	0.85**	(0.04)	0.88*	(0.05)
Labour market status								
Never worked	1.00	–	1.00	–	1.00	–	1.00	–
Unemployed	1.08	(0.06)	1.05	(0.06)	1.04	(0.05)	1.03	(0.05)
Employed	0.85**	(0.05)	0.89*	(0.05)	0.90*	(0.04)	0.95	(0.04)
Material deprivation	1.15***	(0.02)	1.14***	(0.02)	1.18***	(0.01)	1.17***	(0.01)
Subjective social status	0.72***	(0.01)	0.74***	(0.01)	0.72***	(0.01)	0.74***	(0.01)
Cannot afford fish, meat or chicken	1.81***	(0.07)	1.75***	(0.07)	1.93***	(0.07)	1.85***	(0.06)
Distrust in strangers	–	–	1.71***	(0.09)	–	–	1.49***	(0.06)
Socialising	–	–	0.93***	(0.02)	–	–	0.92***	(0.01)
Social comparison								
No comparison	–	–	1.00	–	–	–	1.00	–
Friends and neighbours	–	–	1.06	(0.07)	–	–	0.98	(0.05)
Other	–	–	1.19**	(0.07)	–	–	1.12*	(0.06)
Bad self-rated health	–	–	1.97***	(0.11)	–	–	1.98***	(0.09)
Country-fixed effects	Yes		Yes		Yes		Yes	
AIC	20,862.65		20,328.36		27,037.88		26,401.15	
BIC	21,262.19		20,767.22		27,449.29		26,853.19	
Pseudo R^2^	0.168		0.180		0.170		0.181	
Observations	21,577		21,577		27,415		27,415	

*Source* Authors’ calculations based on data from LITS III (2016)

* *p* < 0.05, ** *p* < 0.01, *** *p* < 0.001

### Treatment estimators of the effect of BMI on dissatisfaction with life

To address the selection bias into specific BMI categories, we calculated average treatment effects (ATEs) from observational data by using IPWRA estimators. Diagnostic statistics, reported in the supplementary materials, Table S4, demonstrate that the absolute majority of coefficients are balanced over treatment groups. The results presented in Table 2 suggest that overweight men have 11% lower dissatisfaction with life than the mean level for men with normal BMI scores. On the other hand, women who are overweight and obese have, respectively, 5% and 9% higher dissatisfaction with life than the mean level for women with normal BMI scores. The full results of IPWRA estimators with auxiliary-equation output are shown in the supplementary materials, Table S5.

**Table 2:** Average treatment effect (ATE) as a percentage of the mean value of dissatisfaction with life from inverse-probability weighting regression adjustment (IPWRA) estimators

	Men		Women	
	ATE	(CI95%)	ATE	(CI95%)
Underweight vs Normal	−0.05	(−0.25,0.15)	0.06	(−0.08,0.19)
Overweight vs Normal	−0.11	(−0.15,-0.06)	0.05	(0.01,0.10)
Obese vs Normal	0.03	(−0.02,0.09)	0.09	(0.04,0.14)

*Source* Authors’ calculations based on data from LITS

### BMI and social comparisons

**Fig. 2:**
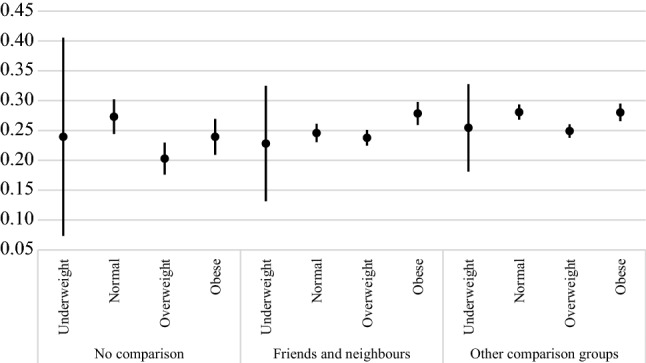
BMI, socioeconomic comparison groups, and dissatisfaction with life in the pooled sample of 34 societies, predictive margins from logistic regressions. *Source* Authors’ calculations based on data from LITS

To understand how social comparison affects the relationship between BMI and dissatisfaction with life, we fit two models for men and women in which the social comparison variable was interacted with the BMI categories. This test does not reveal that social comparison has any moderating effect on the considered relationship among women, but we observe some significant associations for men. In Fig. 2, we plot the predictive margins of BMI categories based on the mode of social comparison made by men. Among those who did not report making any social comparisons, overweight men have the lowest prevalence of dissatisfaction with life; the difference between the normal and obese men is not significant. Yet, among those men who compare their socioeconomic status with friends and neighbours, obese men have significantly higher dissatisfaction with life compared to men who are overweight or have normal BMI.

### Does contextual environment moderate the effect of BMI on dissatisfaction with life?

To test how the contextual factors moderate the effect of BMI on dissatisfaction with life, we fitted multilevel mixed effects logistic regression models with interactions between different BMI categories and contextual variables. First, in Table 3, we use the mean BMI level provided by the WHO, which shows that the national BMI levels do not moderate the effect of the individuals’ own levels of BMI. Nor the extent of the variation in BMI levels within countries, as shown by the standard deviation measure, is related to how BMI affects dissatisfaction with life. In Model 4, nonetheless, when we use the region-specific BMI levels, the interaction coefficient for underweight men and this contextual variable becomes statistically significant. The identified effect of region-specific BMI levels is also significant for women but in a different way. The levels of dissatisfaction with life among obese women are lower in the regions where female obesity is more prevalent.

**Table 3:** Own BMI, mean BMI levels, and dissatisfaction with life in the pooled sample of 34 societies, point estimates from multilevel mixed effects logistic regressions

	Men							
	M1: Interactions with WHO BMI		M2: Interactions with SD BMI		M3: Interactions with cohort BMI		M4: Interactions with region BMI	
	β	(SE)	Β	(SE)	β	(SE)	β	(SE)
*BMI*								
Underweight	0.00	(0.00)	0.37	(0.37)	0.16	(0.18)	0.00^**^	(0.00)
Normal	1.00	–	1.00	–	1.00	–	1.00	–
Overweight	0.12	(0.29)	0.65	(0.24)	0.91	(0.29)	0.58	(0.70)
Obese	0.43	(1.09)	1.59	(0.46)	1.39	(0.32)	2.77	(3.81)
*Contextual factors*								
WHO BMI levels	1.32	(0.33)	–	–	–	–	–	–
Standard deviation of BMI	–	–	0.94	(0.18)	–	–	–	–
Cohort-specific BMI	–	–	–	–	1.01	(0.01)	–	–
Region-specific BMI	–	–	–	–	–	–	1.03	(0.07)
*Interactions terms*								
Underweight	1.31	(0.30)	1.23	(0.29)	1.07	(0.05)	1.60^**^	(0.27)
Normal	1.00	–	1.00	–	1.00	–	1.00	–
Overweight	1.07	(0.09)	1.07	(0.10)	1.00	(0.01)	1.01	(0.05)
Obese	1.03	(0.10)	0.90	(0.07)	0.99	(0.01)	0.96	(0.05)
AIC	19,696.0		20,438.1		20,437.0		20,408.6	
BIC	19,910.6		20,653.5		20,652.4		20,624.0	
Observations	21,577		21,577		21,577		21,577	

	Women							
	M1: Interactions with WHO BMI		M2: Interactions with SD BMI		M3: Interactions with cohort BMI		M4: Interactions with region BMI	
	β	(SE)	β	(SE)	β	(SE)	β	(SE)
*BMI*								
Underweight	2.54	(9.31)	1.57	(0.98)	0.82	(0.32)	1.24	(2.23)
Normal	1.00	–	1.00	–	1.00	–	1.00	–
Overweight	5.75	(9.15)	0.85	(0.22)	1.32	(0.41)	2.07	(1.78)
Obese	3.38	(8.40)	1.20	(0.52)	1.61	(0.42)	12.9^*^	(13.1)
*Contextual factors*								
WHO BMI levels	0.91	(0.17)	–	–	–	–	–	–
Standard deviation of BMI	–	–	1.04	(0.18)	–	–	–	–
Cohort-specific BMI	–	–	–	–	1.01	(0.01)	–	–
Region-specific BMI	–	–	–	–	–	–	1.05	(0.06)
*Interactions terms*								
Underweight	0.97	(0.13)	0.92	(0.12)	1.01	(0.02)	0.99	(0.07)
Normal	1.00	–	1.00	–	1.00	–	1.00	–
Overweight	0.94	(0.06)	1.06	(0.06)	0.99	(0.01)	0.98	(0.03)
Obese	0.96	(0.09)	0.99	(0.09)	0.99	(0.01)	0.91^*^	(0.04)
AIC	25,781.6		26,518.1		26,512.0		26,503.5	
BIC	26,002.8		26,740.0		26,733.9		26,725.4	
Observations	27,415		27,415		27,415		27,415	

*Source* Authors’ calculations based on data from LITS

* *p* < 0.05, ** *p* < 0.01, *** *p* < 0.001

**Fig. 3:**
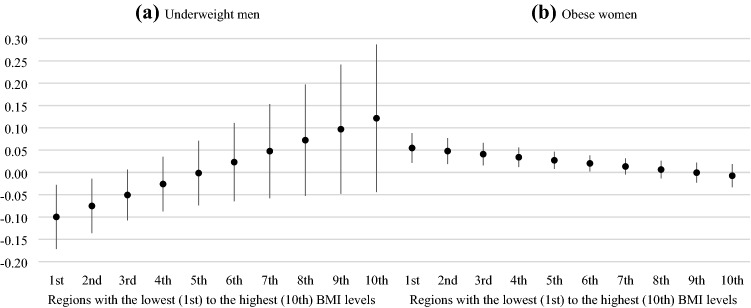
BMI, mean regional BMI level, and dissatisfaction with life in the pooled sample of 34 societies, marginal effects from multilevel mixed effects logistic regressions. *Source* Authors’ calculations based on data from LITS

To see the specific effect of the described contextual variables in Fig. 3, we present the marginal effects of being underweight (for men) or obese (for women) on dissatisfaction with life, in regions with the lowest (1^st^ decile) to the highest (10^th^ decile) prevalence of BMI scores. In two regions with the lowest BMI scores, underweight men have lower levels of dissatisfaction with life. Furthermore, the negative effect of being obese among women does not hold in regions with the top four deciles of BMI distribution. For an illustration, in the region with the lowest level of BMI, obese women have 0.05 (CI95 0.02, 0.09) points higher level of life dissatisfaction than women who are in the healthy BMI category. As Figure S1 in the supplementary materials shows, the observed differences in dissatisfaction with life are driven by both increasing levels of dissatisfaction with life among women with normal BMI and decreasing levels of dissatisfaction with life among obese women in regions with higher overall levels of BMI.

### Additional analysis

To test the robustness of our main findings, we fit regressions with alternative model specifications. First, in Table S6, we use a binary outcome variable denoting whether individuals are satisfied with their lives. Since the prevalence of satisfied individuals is around 50% in the sample, conventional logistic regressions are likely to overestimate the actual associations of the independent variables in the model. On the other hand, the Poisson regressions allow deriving prevalence ratios with corresponding 95% CIs, which are more appropriate measures of association with high prevalence of positive outcomes in the binary-dependent variables [45]. We also fit ordered logistic regression with 5-point Likert scale life satisfaction as the outcome variable. In Table S7, we further derive alternative treatment estimators by accounting for only regression adjustment or inverse-probability weighting. The findings from these auxiliary tests reveal that using binary and ordered outcome, variables and different treatment estimators do not affect the findings reported in the main analysis. In Table S8, we demonstrate that economic development and income inequality do not affect dissatisfaction with life in the considered countries, nor do they moderate the links between BMI and the outcome variable.

## Discussion

In this study, we explored the association between individuals’ BMI category – representing underweight, normal weight, overweight, or obesity – and dissatisfaction with life using a large sample of 48,992 individuals across 34 countries in Europe, the Middle East, and Central Asia. Based on the previous research, we expected that having a higher-than-normal BMI would be associated with higher levels of dissatisfaction with life. Our results demonstrate that that both, higher and lower than normal BMI, were associated with greater prevalence of dissatisfaction with life, and that the association between BMI and dissatisfaction with life differed by gender. Overweight men have lower levels of life dissatisfaction compared to men with normal weight. Conversely, overweight and obese women are more likely to be dissatisfied with life than women with normal weight. We show that overweight men have 11% lower levels of life dissatisfaction compared to men with normal BMI scores, whereas overweight and obese women have, respectively, 5% and 9% higher levels of life dissatisfaction compared to women with normal BMI. The findings were confirmed with treatment effect estimators which are more likely to mitigate selection bias into different BMI categories that conventional regression models do not capture.

BMI has previously been criticised for not being a reliable measure of adiposity and that it performs differently for men and women [46]. Furthermore, for many men, having a larger body weight – and technically being overweight – may be a desirable feature and may, in fact, indicate having a greater muscle mass, not fat [47]. In lay understanding, one is classified as being overweight or obese, not based on their weight, but their appearance. Many men distance themselves from the biomedical definitions of “a healthy weight” and are not even willing to achieve it. It is quite likely that many who were classified as overweight in our study would not see their weight as a reason for frustration or shame. As opposed to that, both underweight and obese men were reported to have experienced weight stigma [48]. The former were also more likely to experience anxiety compared to normal-weight men [49]. The present study shows that they are also more likely to be dissatisfied with their lives.

The situation is very different among women for whom, contrary to men, a smaller body size, regardless of its composition, is generally more socially desirable [50]. Women display greater adherence to social norms including those pertaining to body weight [51]. Female bodies are also subject to overall greater social scrutiny [52]. Being overweight or obese runs counter to the message of healthiness propagated in popular culture [52]. Women with higher-than-normal weight are particularly likely to experience stress and negative emotions in environments where their overweight status is visible through everyday social interactions [53]. This factor was not accounted for in our models, but it is worth investigating in future research.

Based on the insights from the social comparison theory, we also expected that individuals’ patterns of comparison would affect the association between BMI and dissatisfaction with life. Although we did not find that the reference category in the socioeconomic comparison affected the relationship between BMI and dissatisfaction with life for women, the mode of comparison mattered for men. Overweight men who were not making any social comparisons had the lowest levels of life dissatisfaction, whereas obese men comparing their situation to their friends and neighbours had the highest levels of life dissatisfaction among men across all BMI categories. We also investigated how social comparison through social context in which individuals live moderates the relationship between BMI and dissatisfaction with life. We found that the prevalence of obesity in a region where individuals live influences the relationship between BMI and dissatisfaction with life for both genders but in different ways. In the regions with low BMI scores, underweight men have lower levels of dissatisfaction with life compared to underweight men in regions with high BMI scores. With regard to obese women, the effect of obesity on dissatisfaction with life was not observed in regions with overall high BMI scores. Conversely, dissatisfaction with life was more prevalent among obese women living in regions with low BMI scores.

Our findings make a novel contribution to relevant literature. Although being underweight, overweight, or obese has been associated with the levels of life satisfaction in earlier studies, we add additional nuance to the existing knowledge by showing that the association between BMI and being dissatisfied with life is, at least partially, moderated by the contextual environment, and that the character of the contextual effects differs by gender. These findings, along with the earlier evidence from the United States [17], point to the importance of the social context and raise questions about what the BMI categories represent for men and women in different settings and across time and space.

The moderating effect of BMI levels in the region where individuals live can also be interpreted in terms of the “relative obesity”. The concept of relative obesity was proposed by Wadsworth and Pendergast to describe the effect of “an inherently social process connecting obesity to lower rates of subjective wellbeing” [17] (p. 196). Drawing on the social comparison and reference group theories they have deduced that, as is the case of income, the negative or positive effect of a particular trait depends on its levels, among others, whom individuals compare themselves to. Our study supports this assumption with regards to the effect of the regional BMI levels.

One of the limitations of this study is that the social comparison variable was limited to comparison by socioeconomic status and not by the BMI levels in the reference group. To understand specific mechanisms related to social comparison, future studies should account for reference groups’ mean BMI levels. The previous analysis of randomly selected primary sampling units using the same dataset suggests that reported and measured height estimates are not significantly different from each other [36], but individuals, and particularly women, might still underreport their weight [54]. Furthermore, at a macro level, we were able to generate aggregated BMI data only for regional level within the analysed countries, but it might be the case that more localised BMI context (at municipality or even at neighbourhood level) might matter more for individuals. In addition, since the data were cross sectional, we were unable to investigate the chronology in the relation between outcome and risk factor, and hence, we do not claim that we identified a causal relationship between BMI and dissatisfaction with life. Despite using treatment estimators with observational data, IPWRA, it is still possible that an individual’s dissatisfaction with life has a direct or indirect impact on their BMI considering that both the assumption of no unmeasured confounders and the assumption of experimentation in the assignment of treatment are frequently violated in observational samples [55, 56]. At last, since there are important cultural differences in how people understand life satisfaction, our results might be affected by the problem of comparability of answers for subjective questions using large cross-national datasets such as LITS.

Notwithstanding these limitations, this study contributes to our understanding of the contextual dimension of the links between BMI and dissatisfaction with life. Our findings also bear practical implications. Increasing mean BMI levels may result in a weakening association between BMI and life satisfaction including the link between having a higher–than-normal BMI and dissatisfaction with life. Relatedly, declining socioemotional costs of being overweight or obese may further exuberate the obesity epidemic.

## Supplementary Information

Below is the link to the electronic supplementary material.

Supplementary file1 (DOCX 82 KB)

## Data Availability

This study uses publicly available dataset.

## Code availability

STATA code for this study is available from the authors upon request.
