# Supplementary material for: BMI and dissatisfaction with life: contextual factors and socioemotional costs of obesity
Source: Qual Life Res. 2021 Jun 21;31(4):1167–77. doi: 10.1007/s11136-021-02912-3 (PMC8960616; doi:10.1007/s11136-021-02912-3)
Supplement: Supplementary file 1 — Supplementary file1 (DOCX 82 KB) [file 11136_2021_2912_MOESM1_ESM.docx]

Table S1: Profile of respondents by country, gender, and age

|  | *Gender* | | *Age* | | | | Total number |
| --- | --- | --- | --- | --- | --- | --- | --- |
|  | Men | Women | 18-24 | 25-39 | 40-64 | 65+ |  |
| Armenia | 33.40 | 66.60 | 8.06 | 26.72 | 43.68 | 21.55 | 1,527 |
| Belarus | 41.89 | 58.11 | 6.58 | 30.32 | 44.28 | 18.82 | 1,504 |
| Bosnia and Herzegovina | 49.83 | 50.17 | 11.07 | 28.15 | 46.23 | 14.54 | 1,499 |
| Bulgaria | 46.00 | 54.00 | 5.53 | 20.00 | 44.53 | 29.93 | 1,500 |
| Croatia | 45.84 | 54.16 | 7.98 | 25.48 | 45.78 | 20.76 | 1,503 |
| Cyprus | 42.60 | 57.40 | 5.53 | 19.80 | 38.87 | 35.80 | 1,500 |
| Czech Republic | 44.84 | 55.16 | 5.74 | 25.91 | 43.15 | 25.20 | 1,532 |
| Estonia | 36.86 | 63.14 | 4.46 | 15.30 | 41.45 | 38.79 | 1,503 |
| North Macedonia | 48.43 | 51.57 | 9.94 | 25.02 | 41.23 | 23.82 | 1,499 |
| Georgia | 36.94 | 63.06 | 5.70 | 22.75 | 46.09 | 25.46 | 1,508 |
| Germany | 56.27 | 43.73 | 7.40 | 37.20 | 44.93 | 10.47 | 1,500 |
| Greece | 43.78 | 56.22 | 7.19 | 23.89 | 43.78 | 25.15 | 1,503 |
| Hungary | 44.24 | 55.76 | 5.66 | 21.32 | 40.31 | 32.71 | 1,501 |
| Italy | 48.97 | 51.03 | 5.13 | 25.58 | 45.24 | 24.05 | 1,501 |
| Kazakhstan | 35.22 | 64.78 | 7.84 | 34.42 | 47.24 | 10.50 | 1,505 |
| Kosovo | 49.00 | 51.00 | 14.13 | 31.27 | 42.93 | 11.67 | 1,500 |
| Kyrgyz Republic | 46.33 | 53.67 | 12.27 | 32.40 | 45.20 | 10.13 | 1,500 |
| Latvia | 37.33 | 62.67 | 5.73 | 20.13 | 38.60 | 35.53 | 1,500 |
| Lithuania | 39.84 | 60.16 | 7.33 | 18.92 | 41.44 | 32.31 | 1,501 |
| Moldova | 46.03 | 53.97 | 7.28 | 25.20 | 44.38 | 23.15 | 1,512 |
| Mongolia | 44.53 | 55.47 | 9.33 | 37.20 | 44.13 | 9.33 | 1,500 |
| Montenegro | 47.44 | 52.56 | 11.71 | 30.21 | 41.12 | 16.97 | 1,503 |
| Poland | 40.87 | 59.13 | 4.67 | 30.67 | 40.87 | 23.80 | 1,500 |
| Romania | 41.93 | 58.07 | 5.89 | 22.69 | 40.87 | 30.56 | 1,512 |
| Russia | 38.09 | 61.91 | 9.29 | 33.58 | 41.80 | 15.33 | 1,507 |
| Serbia | 47.61 | 52.39 | 6.37 | 25.33 | 43.37 | 24.93 | 1,508 |
| Slovak Republic | 42.03 | 57.97 | 6.54 | 20.14 | 45.27 | 28.04 | 1,544 |
| Slovenia | 48.43 | 51.57 | 5.26 | 16.92 | 43.37 | 34.44 | 1,501 |
| Tajikistan | 46.16 | 53.84 | 16.16 | 35.96 | 41.13 | 6.75 | 1,510 |
| Turkey | 51.27 | 48.73 | 11.93 | 50.27 | 35.13 | 2.67 | 1,500 |
| Ukraine | 38.02 | 61.98 | 4.71 | 27.80 | 46.52 | 20.97 | 1,507 |

*Source:* Authors’ calculations based on data from LITS

Table S2: BMI and dissatisfaction with life in the pooled sample of 34 societies, point estimates from logistic regressions using multiply imputed data-set

|  | *Men* | | | | *Women* | | | |
| --- | --- | --- | --- | --- | --- | --- | --- | --- |
|  | Model 1 | | Model 2 | | Model 1 | | Model 2 | |
|  | β | (SE) | β | (SE) | β | (SE) | β | (SE) |
| Intercept | 1.16 | (0.24) | 1.15 | (0.27) | 0.36*** | (0.07) | 0.40*** | (0.08) |
| BMI |  |  |  |  |  |  |  |  |
| Underweight | 0.98 | (0.17) | 0.86 | (0.15) | 1.07 | (0.10) | 1.03 | (0.10) |
| Normal | 1.00 | ––– | 1.00 | ––– | 1.00 | ––– | 1.00 | ––– |
| Overweight | 0.84*** | (0.03) | 0.85*** | (0.04) | 1.10* | (0.04) | 1.10* | (0.04) |
| Obese | 1.06 | (0.05) | 1.06 | (0.05) | 1.16*** | (0.04) | 1.12** | (0.04) |
| Socio-demographics |  |  |  |  |  |  |  |  |
| Age | 1.03*** | (0.01) | 1.03*** | (0.01) | 1.05*** | (0.01) | 1.05*** | (0.01) |
| Age^2^ | 1.00*** | (0.00) | 1.00*** | (0.00) | 1.00*** | (0.00) | 1.00*** | (0.00) |
| Settlement |  |  |  |  |  |  |  |  |
| Rural | 1.00 | ––– | 1.00 | ––– | 1.00 | ––– | 1.00 | ––– |
| Urban | 0.90** | (0.03) | 0.90** | (0.03) | 0.91** | (0.03) | 0.91** | (0.03) |
| Marital status |  |  |  |  |  |  |  |  |
| Single | 0.70*** | (0.05) | 0.72*** | (0.06) | 0.87* | (0.06) | 0.86* | (0.06) |
| Married | 0.64*** | (0.04) | 0.67*** | (0.05) | 0.73*** | (0.04) | 0.73*** | (0.04) |
| Widowed | 0.78** | (0.07) | 0.79* | (0.08) | 0.86* | (0.05) | 0.87* | (0.05) |
| Divorced | 1.00 | ––– | 1.00 | ––– | 1.00 | ––– | 1.00 | ––– |
| Education |  |  |  |  |  |  |  |  |
| Primary | 1.00 | ––– | 1.00 | ––– | 1.00 | ––– | 1.00 | ––– |
| Secondary | 0.86*** | (0.04) | 0.87** | (0.04) | 0.94 | (0.04) | 0.97 | (0.04) |
| Tertiary | 0.74*** | (0.04) | 0.76*** | (0.04) | 0.86** | (0.04) | 0.90* | (0.05) |
| Labour market status |  |  |  |  |  |  |  |  |
| Never worked | 1.00 | ––– | 1.00 | ––– | 1.00 | ––– | 1.00 | ––– |
| Unemployed | 1.09 | (0.06) | 1.07 | (0.06) | 1.04 | (0.05) | 1.04 | (0.05) |
| Employed | 0.86** | (0.05) | 0.91 | (0.05) | 0.91* | (0.04) | 0.95 | (0.04) |
| Material deprivation | 1.15*** | (0.02) | 1.15*** | (0.02) | 1.18*** | (0.01) | 1.17*** | (0.01) |
| Subjective social status | 0.72*** | (0.01) | 0.74*** | (0.01) | 0.72*** | (0.01) | 0.74*** | (0.01) |
| Cannot afford fish, meat or chicken | 1.80*** | (0.07) | 1.73*** | (0.07) | 1.91*** | (0.06) | 1.83*** | (0.06) |
| Distrust in strangers | ––– | ––– | 1.72*** | (0.09) | ––– | ––– | 1.49*** | (0.06) |
| Socializing | ––– | ––– | 0.93*** | (0.02) | ––– | ––– | 0.92*** | (0.01) |
| Social comparison |  |  |  |  |  |  |  |  |
| No comparison | ––– | ––– | 1.00 | ––– | ––– | ––– | 1.00 | ––– |
| Friends and neighbours | ––– | ––– | 1.05 | (0.07) | ––– | ––– | 1.00 | (0.05) |
| Other | ––– | ––– | 1.17* | (0.07) | ––– | ––– | 1.14* | (0.06) |
| Bad self-rated health | ––– | ––– | 1.98*** | (0.11) | ––– | ––– | 1.96*** | (0.09) |
| Country fixed effects | Yes |  | Yes |  | Yes |  | Yes |  |
| Imputations | 10 |  | 10 |  | 10 |  | 10 |  |
| Observations | 22,500 |  | 22,500 |  | 28,706 |  | 28,706 |  |

*Source:* Authors’ calculations based on data from LITS III (2016)

Table S3: Descriptive statistics of predictors of dissatisfaction with life

|  | *Men* | | *Women* | |
| --- | --- | --- | --- | --- |
|  | % / proportion | SD | % /proportion | SD |
| Age (18-35) | 47.3 | 16.8 | 49.0 | 17.8 |
| Settlement |  |  |  |  |
| Rural | 44.7 | ––– | 41.2 | ––– |
| Urban | 55.3 | ––– | 58.8 | ––– |
| Marital status |  |  |  |  |
| Single | 21.8 | ––– | 15.6 | ––– |
| Married | 65.2 | ––– | 54.0 | ––– |
| Widowed | 6.2 | ––– | 19.7 | ––– |
| Divorced | 6.8 | ––– | 10.7 | ––– |
| Education |  |  |  |  |
| Primary | 25.5 | ––– | 27.3 | ––– |
| Secondary | 51.3 | ––– | 48.2 | ––– |
| Tertiary | 23.2 | ––– | 24.5 | ––– |
| Labour market status |  |  |  |  |
| Never worked | 16.1 | ––– | 26.3 | ––– |
| Unemployed | 25.6 | ––– | 32.0 | ––– |
| Employed | 58.3 | ––– | 41.7 | ––– |
| Material deprivation (0-7) | 0.75 | 1.4 | 0.88 | 1.5 |
| Subjective social status (1-10) | 4.6 | 1.7 | 4.5 | 1.7 |
| Cannot afford fish, meat or chicken (0-1) | 0.32 | 0.46 | 0.34 | 0 .48 |
| Distrust in strangers (0-1) | 0.12 | 0.33 | 0.14 | 0.35 |
| Socializing (1-5) | 3.60 | 1.00 | 3.54 | 1.01 |
| Social comparison |  |  |  |  |
| No comparison | 10.8 | ––– | 12.5 | ––– |
| Friends and neighbours | 34.5 | ––– | 33.9 | ––– |
| Other | 54.7 | ––– | 53.5 | ––– |
| Bad self-rated health (0-1) | 0.10 | 0.30 | 0.14 | 0.35 |

*Source:* Authors’ calculations based on data from LITS

Table S4: IPWRA diagnostic statistics to check for covariate balance over treatment groups

|  | *Men* | | | | | | *Women* | | | | | |
| --- | --- | --- | --- | --- | --- | --- | --- | --- | --- | --- | --- | --- |
|  | Normal BMI vs Underweight | | Normal BMI vs overweight | | Normal BMI vs obese | | Normal BMI vs Underweight | | Normal BMI vs overweight | | Normal BMI vs obese | |
|  | Standardized differences | | Standardized differences | | Standardized differences | | Standardized differences | | Standardized differences | | Standardized differences | |
|  | Raw | Weighted | Raw | Weighted | Raw | Weighted | Raw | Weighted | Raw | Weighted | Raw | Weighted |
| Age | -0.13 | -0.02 | 0.30 | 0.00 | 0.43 | 0.00 | -0.54 | -0.03 | 0.58 | 0.00 | 0.59 | -0.01 |
| Age^2^ | -0.09 | -0.02 | 0.26 | 0.00 | 0.38 | 0.00 | -0.45 | -0.03 | 0.53 | 0.00 | 0.56 | -0.01 |
| Settlement |  |  |  |  |  |  |  |  |  |  |  |  |
| Urban | 0.07 | 0.02 | 0.01 | 0.01 | 0.02 | 0.01 | -0.05 | 0.01 | 0.04 | 0.01 | 0.06 | -0.01 |
| Marital status |  |  |  |  |  |  |  |  |  |  |  |  |
| Single | 0.19 | 0.03 | -0.24 | -0.01 | -0.30 | 0.00 | 0.42 | 0.04 | -0.34 | 0.00 | -0.29 | 0.00 |
| Married | -0.14 | 0.02 | 0.20 | 0.00 | 0.22 | 0.00 | -0.24 | -0.04 | 0.06 | 0.00 | -0.02 | 0.00 |
| Widowed | -0.09 | -0.04 | 0.02 | 0.00 | 0.07 | 0.00 | -0.20 | -0.01 | 0.27 | 0.00 | 0.34 | 0.00 |
| Education |  |  |  |  |  |  |  |  |  |  |  |  |
| Secondary | 0.10 | 0.02 | -0.01 | 0.00 | -0.01 | -0.01 | -0.02 | 0.01 | -0.01 | 0.01 | 0.00 | 0.00 |
| Tertiary | -0.08 | -0.05 | -0.02 | 0.00 | -0.10 | 0.00 | 0.10 | 0.00 | -0.19 | -0.01 | -0.23 | 0.00 |
| Labour market status |  |  |  |  |  |  |  |  |  |  |  |  |
| Unemployed | 0.21 | -0.05 | 0.11 | 0.00 | 0.21 | 0.01 | -0.14 | 0.02 | 0.25 | 0.01 | 0.30 | 0.00 |
| Employed | -0.34 | 0.02 | 0.06 | 0.00 | -0.11 | 0.00 | -0.10 | -0.02 | -0.22 | 0.00 | -0.30 | 0.00 |
| Material deprivation | 0.38 | 0.02 | -0.18 | -0.01 | -0.05 | 0.01 | 0.09 | 0.07 | 0.01 | 0.00 | 0.13 | -0.01 |
| Meat no | 0.16 | -0.03 | -0.10 | -0.01 | -0.07 | 0.00 | 0.05 | 0.10 | 0.09 | 0.01 | 0.15 | -0.01 |
| Subjective social status | -0.27 | 0.04 | 0.08 | 0.00 | 0.04 | 0.00 | 0.01 | -0.03 | -0.14 | 0.00 | -0.18 | 0.01 |
| Hours watching TV | 0.12 | -0.05 | 0.08 | 0.00 | 0.13 | -0.01 | -0.13 | 0.03 | 0.19 | 0.01 | 0.21 | 0.00 |

*Source:* Authors’ calculations based on data from LITS

Table S5: IPWRA auxiliary equation output for treatment models

|  | *Men* | | | | | | *Women* | | | | | |
| --- | --- | --- | --- | --- | --- | --- | --- | --- | --- | --- | --- | --- |
|  | Normal BMI vs Underweight | | Normal BMI vs overweight | | Normal BMI vs obese | | Normal BMI vs Underweight | | Normal BMI vs overweight | | Normal BMI vs obese | |
|  | β | (SE) | β | (SE) | β | (SE) | β | (SE) | β | (SE) | β | (SE) |
| Intercept |  |  |  |  |  |  |  |  |  |  |  |  |
| Age | 0.99 | -0.01 | 1.02*** | 0.00 | 1.02*** | 0.00 | 0.97*** | 0.00 | 1.03*** | 0.00 | 1.03*** | 0.00 |
| Settlement |  |  |  |  |  |  |  |  |  |  |  |  |
| Rural | 1.00 | ––– | 1.00 | ––– | 1.00 | ––– | 1.00 | ––– | 1.00 | ––– | 1.00 | ––– |
| Urban | 1.12 | -0.19 | 1.06 | -0.04 | 0.99 | -0.04 | 0.99 | -0.08 | 1.08* | -0.04 | 1.04 | -0.04 |
| Marital status |  |  |  |  |  |  |  |  |  |  |  |  |
| Single | 1.57 | -0.53 | 0.91 | -0.07 | 0.86 | -0.07 | 1.29 | -0.19 | 0.80*** | -0.05 | 0.99 | -0.07 |
| Married | 0.90 | -0.31 | 1.29*** | -0.09 | 1.26** | -0.10 | 0.74* | -0.10 | 1.26*** | -0.07 | 1.21*** | -0.07 |
| Widowed | 0.81 | -0.39 | 1.00 | -0.10 | 0.95 | -0.10 | 1.00 | -0.20 | 1.10 | -0.07 | 1.22** | -0.08 |
| Divorced | 1.00 | ––– | 1.00 | ––– | 1.00 | ––– | 1.00 | ––– | 1.00 | ––– | 1.00 | ––– |
| Education |  |  |  |  |  |  |  |  |  |  |  |  |
| Primary | 1.00 | ––– | 1.00 | ––– | 1.00 | ––– | 1.00 | ––– | 1.00 | ––– | 1.00 | ––– |
| Secondary | 1.01 | -0.21 | 1.13** | -0.05 | 0.99 | -0.05 | 0.88 | -0.10 | 0.97 | -0.04 | 0.82*** | -0.03 |
| Tertiary | 0.93 | -0.24 | 1.03 | -0.05 | 0.80*** | -0.05 | 1.00 | -0.12 | 0.79*** | -0.04 | 0.61*** | -0.03 |
| Labour market status |  |  |  |  |  |  |  |  |  |  |  |  |
| Never worked | 1.00 | ––– | 1.00 | ––– | 1.00 | ––– | 1.00 | ––– | 1.00 | ––– | 1.00 | ––– |
| Unemployed | 1.29 | -0.28 | 1.41*** | -0.08 | 1.06 | -0.07 | 0.89 | -0.11 | 1.03 | -0.05 | 1.00 | -0.05 |
| Employed | 0.64* | -0.13 | 1.60*** | -0.08 | 1.06 | -0.06 | 0.64*** | -0.07 | 1.07 | -0.05 | 0.95 | -0.04 |
| Material deprivation | 1.10 | -0.06 | 0.94*** | -0.01 | 1.00 | -0.02 | 1.03 | -0.03 | 0.97* | -0.01 | 1.01 | -0.01 |
| Meat no | 0.83 | -0.14 | 0.90** | -0.04 | 0.86** | -0.04 | 1.07 | -0.10 | 1.04 | -0.04 | 1.04 | -0.04 |
| Subjective social status | 0.90 | -0.05 | 1.03** | -0.01 | 1.03** | -0.01 | 1.00 | -0.03 | 0.99 | -0.01 | 0.99 | -0.01 |
| Hours watching TV | 1.04 | -0.04 | 1.02 | -0.01 | 1.02* | -0.01 | 0.97 | -0.02 | 1.04*** | -0.01 | 1.04*** | -0.01 |
| Country fixed effects | Yes |  | Yes |  | Yes |  | Yes |  | Yes |  | Yes |  |
| Observations | 21,577 |  | 21,577 |  | 21,577 |  | 27,415 |  | 27,415 |  | 27,415 |  |

*Source:* Authors’ calculations based on data from LITS

Table S6: BMI and dissatisfaction with life in the pooled sample of 34 societies, prevalence ratios (PR) and odds ratios (OR) from Poisson and ordered logistic regressions

|  | *Satisfied with life* | | | | *Life satisfaction* | | | |
| --- | --- | --- | --- | --- | --- | --- | --- | --- |
|  | Men | | Women | | Men | | Women | |
|  | Model 1: Poisson | | Model 2: Poisson | | Model 1: Ordered logit | | Model 2: Ordered logit | |
|  | RR | (SE) | RR | (SE) | OR | (SE) | OR | (SE) |
| Intercept | 0.28*** | (0.02) | 0.37*** | (0.03) |  |  |  |  |
| Cutting point 1 | ––– | ––– | ––– | ––– | 0.25*** | (0.04) | 0.12*** | (0.02) |
| Cutting point 2 | ––– | ––– | ––– | ––– | 1.19 | (0.21) | 0.58*** | (0.09) |
| Cutting point 3 | ––– | ––– | ––– | ––– | 4.42*** | (0.77) | 2.06*** | (0.30) |
| Cutting point 4 | ––– | ––– | ––– | ––– | 68.7*** | (12.19) | 31.2*** | (4.69) |
| BMI |  |  |  |  |  |  |  |  |
| Underweight | 1.09 | (0.07) | 0.96 | (0.03) | 1.10 | (0.16) | 0.93 | (0.07) |
| Normal | 1.00 | ––– | 1.00 | ––– |  |  |  |  |
| Overweight | 1.05*** | (0.02) | 0.95*** | (0.01) | 1.09** | (0.03) | 0.88*** | (0.02) |
| Obese | 1.01 | (0.02) | 0.95*** | (0.01) | 0.97 | (0.03) | 0.87*** | (0.03) |
| Socio-demographics |  |  |  |  |  |  |  |  |
| Age | 0.98*** | (0.00) | 0.98*** | (0.00) | 0.97*** | (0.00) | 0.95*** | (0.00) |
| Age^2^ | 1.00*** | (0.00) | 1.00*** | (0.00) | 1.00*** | (0.00) | 1.00*** | (0.00) |
| Settlement |  |  |  |  |  |  |  |  |
| Rural | 1.00 | ––– | 1.00 | ––– |  |  |  |  |
| Urban | 1.05*** | (0.01) | 1.05*** | (0.01) | 1.09** | (0.03) | 1.11*** | (0.03) |
| Marital status |  |  |  |  |  |  |  |  |
| Single | 1.16*** | (0.04) | 1.09*** | (0.03) | 1.33*** | (0.08) | 1.23*** | (0.06) |
| Married | 1.24*** | (0.04) | 1.17*** | (0.03) | 1.48*** | (0.08) | 1.34*** | (0.05) |
| Widowed | 1.11* | (0.05) | 1.03 | (0.03) | 1.21** | (0.09) | 1.08 | (0.05) |
| Divorced | 1.00 | ––– | 1.00 | ––– |  |  |  |  |
| Education |  |  |  |  |  |  |  |  |
| Primary | 1.00 | ––– | 1.00 | ––– |  |  |  |  |
| Secondary | 1.05** | (0.02) | 1.06*** | (0.02) | 1.09** | (0.04) | 1.05 | (0.03) |
| Tertiary | 1.13*** | (0.02) | 1.11*** | (0.02) | 1.32*** | (0.05) | 1.17*** | (0.04) |
| Labour market status |  |  |  |  |  |  |  |  |
| Never worked | 1.00 | ––– | 1.00 | ––– |  |  |  |  |
| Unemployed | 0.98 | (0.02) | 1.01 | (0.02) | 0.97 | (0.04) | 1.01 | (0.03) |
| Employed | 1.04* | (0.02) | 1.02 | (0.02) | 1.10* | (0.05) | 1.04 | (0.03) |
| Material deprivation | 0.95*** | (0.01) | 0.93*** | (0.01) | 0.89*** | (0.01) | 0.87*** | (0.01) |
| Subjective social status | 1.12*** | (0.00) | 1.13*** | (0.00) | 1.34*** | (0.01) | 1.35*** | (0.01) |
| Mean no | 0.75*** | (0.01) | 0.75*** | (0.01) | 0.56*** | (0.02) | 0.56*** | (0.02) |
| Distrust in strangers | 0.93*** | (0.02) | 0.95** | (0.02) | 0.71*** | (0.03) | 0.77*** | (0.03) |
| Socializing | 1.03*** | (0.01) | 1.04*** | (0.01) | 1.10*** | (0.02) | 1.11*** | (0.01) |
| Social comparison |  |  |  |  |  |  |  |  |
| No comparison | 1.00 | ––– | 1.00 | ––– |  |  |  |  |
| Friends and neighbours | 0.98 | (0.02) | 0.98 | (0.02) | 0.93 | (0.04) | 0.96 | (0.04) |
| Other | 0.97 | (0.02) | 0.98 | (0.02) | 0.90* | (0.04) | 0.93 | (0.04) |
| Bad self-rated health | 0.70*** | (0.02) | 0.68*** | (0.02) | 0.54*** | (0.03) | 0.52*** | (0.02) |
| Country fixed effects | Yes |  | Yes |  | Yes |  | Yes |  |
| AIC | 34426 |  | 43306 |  | 55667.60 |  | 70841.70 |  |
| BIC | 34865 |  | 43758 |  | 56130.40 |  | 71318.40 |  |
| Observations | 21577 |  | 27415 |  | 21577 |  | 27415 |  |

*Source:* Authors’ calculations based on data from LITS

Table S7: Average treatment effect (ATE) as a percentage of the mean value of dissatisfaction with life from

regression adjustment and inverse-probability weighting (IPW) estimators

|  | *Regression adjustment* | | | |
| --- | --- | --- | --- | --- |
|  | Males |  | Females |  |
|  | ATE | (CI95%) | ATE | (CI95%) |
| Normal vs underweight | 0.08 | (-0.17,0.33) | 0.11 | (-0.04,0.26) |
| Normal vs overweight | -0.09 | (-0.14,0.05) | 0.07 | (0.03,0.12) |
| Normal vs obese | 0.04 | (-0.01,0.10) | 0.10 | (0.05.0.15) |
|  | *Inverse-probability weighting* | | | |
|  | Males |  | Females |  |
|  | ATE | (CI95%) | ATE | (CI95%) |
| Normal vs underweight | -0.03 | (-0.28,0.21) | 0.15 | (-0.08,0.39) |
| Normal vs overweight | -0.11 | (-0.16,-0.07) | 0.07 | (0.02,0.12) |
| Normal vs obese | 0.05 | (-0.00,0.11) | 0.11 | (0.06,0.16) |

*Source:* Authors’ calculations based on data from LITS

Table S8: BMI, contextual factors, and dissatisfaction with life in the pooled sample of 34 societies, odds rations from multilevel mixed effects logistic regressions

|  | *Men* | | | | | | *Women* | | | | | |
| --- | --- | --- | --- | --- | --- | --- | --- | --- | --- | --- | --- | --- |
|  | Model 1 |  | Model 2 |  | Model 3 |  | Model 1 |  | Model 2 |  | Model 3 |  |
|  | OR | (SE) | OR | (SE) | OR | (SE) | OR | (SE) | OR | (SE) | OR | (SE) |
| *BMI* |  |  |  |  |  |  |  |  |  |  |  |  |
| Underweight | 0.87 | (0.15) | 0.92 | (0.18) | 0.90 | (0.16) | 1.08 | (0.08) | 1.08 | (0.09) | 1.08 | (0.08) |
| Normal | 1.00 | ––– | 1.00 | ––– | 1.00 | ––– | 1.00 | ––– | 1.00 | ––– | 1.00 | ––– |
| Overweight | 0.85** | (0.05) | 0.85** | (0.05) | 0.85*** | (0.04) | 1.11* | (0.05) | 1.11* | (0.05) | 1.11* | (0.05) |
| Obese | 1.06 | (0.06) | 1.06 | (0.06) | 1.06 | (0.05) | 1.14* | (0.07) | 1.14* | (0.07) | 1.14* | (0.07) |
| *Contextual factors* |  |  |  |  |  |  |  |  |  |  |  |  |
| GDP ppp per capita | 1.05 | (0.20) | 1.01 | (0.20) | 1.05 | (0.15) | 1.09 | (0.19) | 1.07 | (0.20) | 1.09 | (0.19) |
| Gini coefficient | 1.02 | (0.14) | 1.02 | (0.14) | 1.00 | (0.15) | 1.01 | (0.12) | 1.01 | (0.12) | 1.01 | (0.12) |
| *Interactions terms* |  |  |  |  |  |  |  |  |  |  |  |  |
| Underweight | ––– | ––– | 1.25 | (0.24) | 0.79 | (0.15) | ––– | ––– | 0.97 | (0.10) | 0.94 | (0.06) |
| Normal | ––– | ––– | 1.00 | ––– | 1.00 | ––– |  |  | 1.00 | ––– | 1.00 | ––– |
| Overweight | ––– | ––– | 1.07 | (0.06) | 1.03 | (0.04) | ––– | ––– | 1.04 | (0.05) | 0.98 | (0.04) |
| Obese | ––– | ––– | 1.05 | (0.08) | 1.03 | (0.05) | ––– | ––– | 1.00 | (0.07) | 1.03 | (0.07) |
| AIC | 20440.9 |  | 20443.5 |  | 20444.3 |  | 26515.6 |  | 26520.5 |  | 26519.6 |  |
| BIC | 20640.4 |  | 20666.9 |  | 20667.8 |  | 26721.1 |  | 26750.6 |  | 26749.7 |  |
| Observations | 21577 |  | 21577 |  | 21577 |  | 27415 |  | 27415 |  | 27415 |  |

*Source:* Authors’ calculations based on data from LITS

Figure S1: BMI, mean regional levels of BMI, and share of individuals reporting dissatisfaction with life in the pooled sample of 34 societies, predictive margins from mixed effects logistic regressions

|  |  |
| --- | --- |

*Source:* Authors’ calculations based on data from LITS
